# Strategies to improve the efficiency and utility of multidisciplinary team meetings in urology cancer care: a survey study

**DOI:** 10.1186/1472-6963-14-377

**Published:** 2014-09-08

**Authors:** Benjamin W Lamb, Rozh T Jalil, Nick Sevdalis, Charles Vincent, James S A Green

**Affiliations:** Department of Surgery and Cancer, Imperial College London, St. Mary’s Hospital, 5th Floor Medical School Building, London, W2 1PG UK; Department of Psychology, University of Oxford, Oxford, UK; Department of Urology, Whipps Cross University Hospital, London, UK; Department of Health and Social Care, London Southbank University, London, UK

**Keywords:** Urology, Multidisciplinary, Team, Decision-making, Efficacy, Efficiency

## Abstract

**Background:**

The prevalence of multidisciplinary teams (MDT) for the delivery of cancer care is increasing globally. Evidence exists of benefits to patients and healthcare professionals. However, MDT working is time and resource intensive. This study aims to explore members’ views on existing practices of urology MDT working, and to identify potential interventions for improving the efficiency and productivity of the MDT meeting.

**Methods:**

Members of urology MDTs across the UK were purposively recruited to participate in an online survey. Survey items included questions about the utility and efficiency of MDT meetings, and strategies for improving the efficiency of MDT meetings: treating cases by protocol, prioritising cases, and splitting the MDT into subspeciality meetings.

**Results:**

173 MDT members participated (Oncologists n = 77, Cancer Nurses n = 54, Urologists n = 30, other specialities n = 12). 68% of respondents reported that attending the MDT meeting improves efficiency in care through improved clinical decisions, planning investigations, helping when discussing plans with patients, speciality referrals, documentation/patient records. Participants agreed that some cases including low risk, non-muscle invasive bladder cancer and localised, low-grade prostate cancer could be managed by pre-agreed pathways, without full MDT review. There was a consensus that cases at the MDT meeting could be prioritised by complexity, tumour type, or the availability of MDT members. Splitting the MDT meeting was unpopular: potential disadvantages included loss of efficiency, loss of team approach, unavailability of members and increased administrative work.

**Conclusion:**

Key urology MDT members find the MDT meeting useful. Improvements in efficiency and effectiveness may be possible by prioritising cases or managing some low-risk cases according to previously agreed protocols. Further research is needed to test the effectiveness of such strategies on MDT meetings, cancer care pathways and patient outcomes in clinical practice.

## Background

Evidence is emerging of the benefits of multidisciplinary working in cancer care [1]. In the UK multidisciplinary team (MDT) working in cancer care has been mandatory for over a decade, and all cases of new or suspected cancer must be discussed in an MDT meeting [2]. Such teams, which involve surgeons, radiation and medical oncologists, radiologists, pathologists, sometimes specialist nurses (always in the UK) and MDT coordinators, work together and meet regularly (e.g. weekly) to plan investigations and treatment for patients [2].

The concept of MDT-driven care in cancer is recognised internationally, however, its uptake varies. MDT-driven care is more firmly embedded into some healthcare systems (e.g. UK, Australia) than others (e.g. US) – possibly due to a combination of differences in the geography of healthcare institutions (e.g. centralised care approach, driven by cancer centres in the UK versus largely community-based care delivery in the US), financial drivers, and potentially also the nature of the evidence-base supporting MDTs. Some studies have emerged indicating benefits of MDT driven care – overall however the evidence is not conclusive or based on strong research designs. Reviews indicate that overall MDTs do improve the care process, and the largest study that we are aware of to date on breast cancer patients also indicates improved survival [1, 3, 4].

Recent thinking on MDTs has focused on how the teams actually function and how MDT meetings run in practice. Consensus over what constitutes effective MDT working is high, and MDT members remain positive about MDTs [5, 6]. It is thought that time spent at the MDT saves time for members later [7–9]. A key problem in MDT working in settings with high patient volumes is that team meetings are often fast-paced, with large numbers of cases leaving inadequate time for in-depth discussion [10–12]. In addition to the initial MDT discussion of cases of newly diagnosed cancer, many cases will need repeated discussion at the MDT meeting at various points along the care pathway – e.g. when planning or reviewing the results of treatment. Moreover, although there is no obligation to discuss cases of recurrent or relapsing disease, such cases are often complex and thus may benefit most from a MDT approach [13]. With increasing numbers of cancer patients being managed by MDTs, one critique of the current MDT process is that it is time and resource intensive – such that the team-members in attendance do not have adequate time to prepare for the meeting [14]. Further, how best to represent the patient’s interests and views within the meeting remains an area of debate: there is agreement that MDT treatment plan should be explained and fully discussed with patients afterwards, however, most members do not support the inclusion of patients in MDT meetings, arguing that this will increase their anxiety and potentially hinder the case review [14–16].

To address some of the issues above, there is an increasing body of research aiming to evaluate how MDTs function and what the quality of their decision making is [3, 4]. Ultimately, such descriptive studies aim to provide a background for the improvement of MDT working and clinical decision-making in order to ensure that every case receives thorough and comprehensive review [3, 4, 17]. Meeting efficiency features highly in the emerging evidence – in other words, there is increasing interest in how the efficiency of MDT meetings can be improved to allow in depth discussion of cases that require complex decision-making, avoiding inappropriate use of time or resources on simple cases, whilst ensuring that quality assurance is guaranteed for all cases. Proposed solutions include treating simple cases by protocol and approving such treatment plans outside the MDT meeting; prioritisation of cases; and splitting larger, high-volume MDTs into smaller, more specialist and hence more manageable meetings (i.e. individual MDT meetings for prostate, bladder and kidney cancers, instead of a very large ‘urology’ MDT).

This study aims to better understand MDT working and add to the relevant evidence – with a specific focus on the urology MDT. The specific objectives of this study were to assess the perceptions of MDT members regarding:

- The usefulness of MDT meetings
- Whether MDT working saves time later, and if so, how
- Strategies for improving the efficiency of MDT meetings, namely
- Treating some cases by pre-defined protocol, with ratification of management plans by the MDT chair alone
- Prioritising cases at the MDT meeting
- Splitting large MDT meetings into smaller, subspeciality ones

The study was overall descriptive and exploratory in nature.

## Method

### Participants

Two surveys were conducted on separate occasions. The first survey was sent to the attendees of the British Uro-oncology Group (BUG) annual meeting in 2011. The second survey was administered to the attendees of a national Royal Society of Medicine (RSM) meeting that was jointly organised by the Oncology Section of the RSM and BUG in 2012 (‘*What’s new – What’s changing in prostate cancer?’* meeting). Recruitment was purposive in order to ensure representation of key members of the urology MDT, i.e. professional groups who have direct clinical contact with patients: Urological Surgeons, Cancer Nurse Specialists (CNS), and Uro-Oncologists. The survey link was emailed to the attendees of the events prior to the meetings. No reminders were administered, as this was not feasible.

### Study design and materials

This was a prospective cross-sectional study. MDT members were sent an electronic invitation to fill out an electronic survey via freely available software (http://www.surveymonkey.com). The survey included a mix of closed and open ended items. Four questions were answered on a 5-point Likert scale (anchored at 1 = completely disagree, 3 = neither agree nor disagree, 5 = completely agree), six questions were multiple choice, and three questions required free text responses. The survey consisted of questions covering amount of time spent in the MDT meeting; whether respondents thought that all cases are discussed at MDT; whether the MDT meeting saves time later; respondents’ perceived usefulness of the MDT meeting; whether cases could be treated by protocol, and which cases this might apply to; whether cases could be prioritised; and whether the MDT meeting could be split into subspeciality meetings. Questions regarding the demographics of participants were also included.

### Data analyses

Descriptive statistics are reported for each element of the evaluation (median, minimum, maximum; or percentage and 95% confidence intervals). Differences in ratings of professional groups (surgeons, oncologists, nurses) were assessed statistically using the Kruskal Wallis test (KWT). All statistical analyses were performed using SPSS version 20.0 (SPSS Inc., Chicago, IL, USA). Significance was taken at the 0.05 level. A qualitative approach was used to analyse responses to free-text questions: A Grounded Theory approach was applied using a skeleton coding framework where data were coded to primary codes in the framework, and new codes were added as new themes emerged in the data [18]. Themes are presented according to frequency to facilitate reading.

### Ethics

The protocol for the study was reviewed and approved by the Whipps Cross University Hospital Research and Development department. Participation in the study was on the basis of informed consent and the study was carried out in compliance with the Helsinki Declaration.

## Results

### Characteristics of participants

In total, 173 participants completed the survey. The overall response rate was 54% (320 email invitations were sent). Respondents included 77 Consultant Uro- Oncologists (44.5%), 54 Nurses (31.2%), 30 Consultant Urologists (17.3%), and 12 other specialities (6.9%) (General Practitioner = 3, Radiologists = 2, Radiographer = 3, Radiotherapist = 3 and Scientist = 1).

### Time spent at the urology MDT meeting

**Figure 1:**
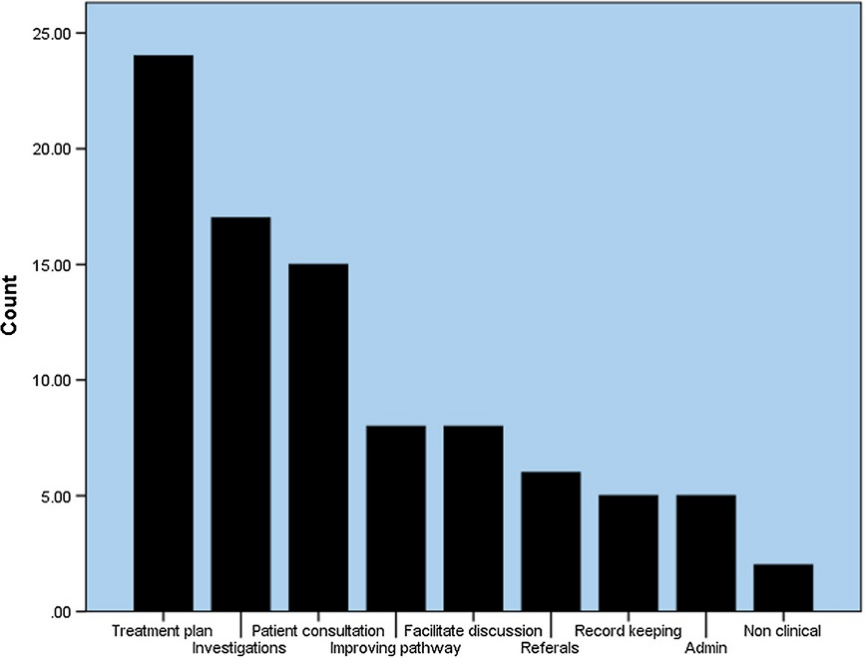
**Figure presenting emergent themes from free-text responses to the question, “How does attending the MDT meeting save time later?”.** Themes are presented according to frequency (N = 58).

The median time per week spent at the urology MDT meeting by Oncologists was 2.0 hours (range 0–6 hours), by Urologists 2.0 hours (1–5 hours) and by Nurses 2.0 hours (0–5 hours) – with Oncologists also spending significantly more time (median 2.0; range 0–6) than Urologists or Nurses (both medians 0.0; range 0–2) in other, non-urology MDTs (P ≤ 0.001; KWT). Sixty-eight per cent (n = 75) of the respondents said that attending MDT meetings saves them time later. Emergent themes from the responses (N = 58) to the question about how specifically the MDT meeting saves time later are presented in Table 1 and Figure 1. Regarding the utility of the MDT meeting, the median proportion of time spent at the MDT meeting that respondents thought was useful for their own patients was 60%, for their colleagues’ patients was 70%, and for themselves was 50%. There were no significant differences between professional groups.

**Table 1:** **Table presenting emergent themes (Left column) from free-text responses to the question, “How does attending the MDT meeting save time later?”**

Theme	Explanation
**Treatment plan**	Plans for treatment can be formulated and clarified at MDT meeting
**Investigations**	Investigations (e.g. radiological investigations) can be collated and reviewed
**Patient consultation**	Being familiar with the clinical history, results of investigations and proposed treatment facilitates consultation with patients
**Improving pathway**	The passage of patients from one clinician to another is quicker and more direct
**Facilitate discussion**	Face to face discussion allows questions to be asked and answered directly
**Referrals**	Inappropriate referrals can be avoided and appropriate referrals made directly in person
**Record keeping**	A single record of results and multidisciplinary discussion can be created
**Admin**	Patient follow-up is streamlined and patients are not lost
**Non-clinical**	Improved relationships between team members

Explanations for the displayed themes are presented in the right column.

### Improving the efficiency of the MDT meeting

**Figure 2:**
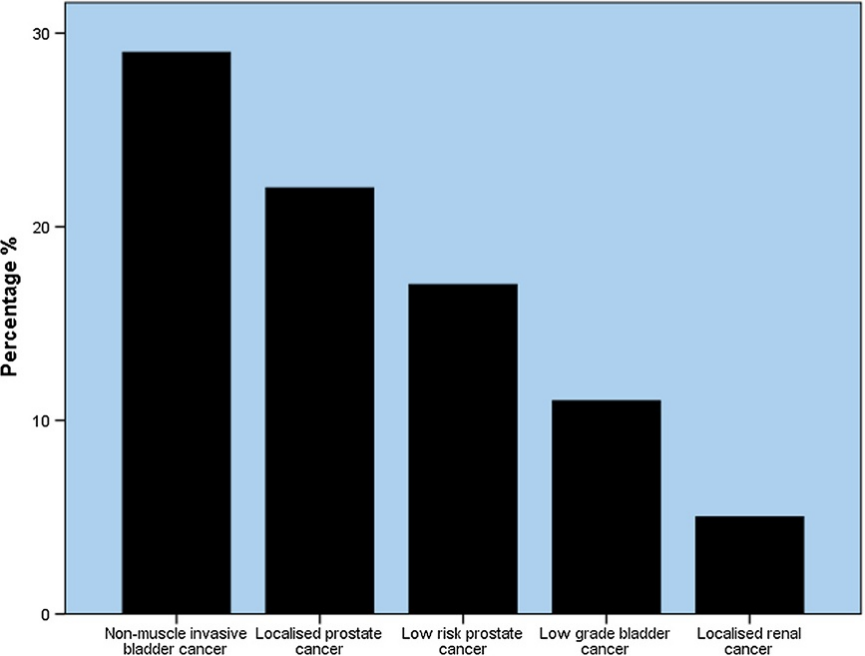
**Figure displaying responses to the question, “What tumour types could be managed without going through MDT meeting discussion?”.** Data displayed according to frequency (N = 60).

Participants ‘agreed’ that at local (within their own hospitals) or specialist (linked to regional cancer centres) MDT meetings, some cases, for which MDT review is currently mandatory, could be treated according to a pre-defined pathway. There was no significant difference between professional groups in this view (*P =* 0.075, 0.328; KWT). Figure 2 displays participants’ views regarding which cases could be treated by pre-approved protocol, rather than being subject to full discussion in the MDT meeting (N = 60).

The majority of respondents agreed that cases at the MDT meeting could be prioritised by tumour type (Yes = 80%), case complexity (Yes = 69.1%), or the availability of MDT members within the meeting (Yes = 61.5%).

**Figure 3:**
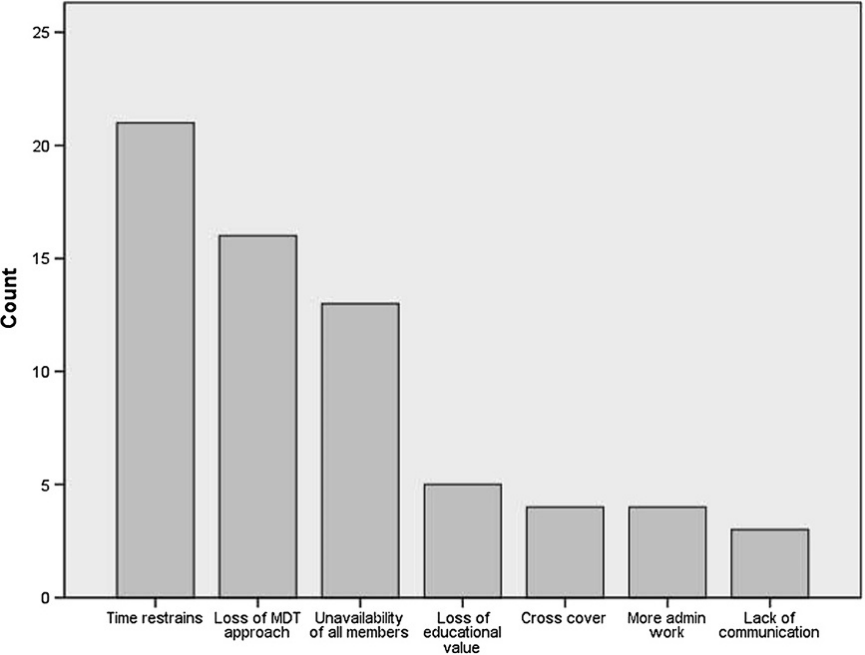
**Figure presenting emergent themes from free-text responses to the question, “What are the potential disadvantages of splitting the MDT meeting?”.** Themes are presented according to frequency (N = 72).

A minority of respondents agreed that the MDT meeting could be split into smaller, subspeciality meetings by tumour type (Yes = 40%), case complexity (Yes = 16.8%), and MDT member availability (Yes = 18.1%). There was a significant difference between professional groups regarding whether the MDT meeting could be split by MDT member availability, with 0.0% of Urologists, 8.7% of CNS and 32.6% of Oncologists in agreement (P = 0.001, KWT). Data relating to participants’ free-text responses (N = 72) to a question about the perceived disadvantages of splitting the MDT meeting are displayed in Table 2 and Figure 3.

**Table 2:** **Table presenting emergent themes (Left column) from free-text responses to the question, “What are the potential disadvantages of splitting the MDT meeting?”**

Theme	Explanation
**Time restraints**	More time consuming to attend greater number of smaller meetings
**Loss of MDT approach**	Fragmentation of cancer care into a number of different subgroups
**Unavailability of all members**	Difficulty scheduling multiple meetings into MDT members’ job plans
**Loss of educational value**	Loss of experience of broad range of tumour types
**Cross cover**	More cover needed for MDT member absence
**More admin work**	Greater amount of record keeping and administrative work load associated with larger number of meetings
**Lack of communication**	Loss of face to face contact with specialists who may have slightly different speciality interest and attend different meeting

Explanations for the displayed themes are presented in the right column.

## Discussion

Our results suggest that attending the MDT meeting is a considerable part of our participants work load, but that they feel that overall their attendance is useful and can save them time later by giving them access to results, plans, discussion with colleagues and smoothing the pathway. Participants felt that certain cases of low risk prostate, bladder and renal cancer could be treated by a predefined protocol and approved at the MDT. Participants responded favourably to suggestions that cases could be prioritised by complexity or tumour type, but prioritisation by team members’ availability was less popular. In contrast, participants generally did not agree that the MDT meeting could be split into subspeciality meetings neither by complexity, tumour type nor by team members’ availability – possibly indicating a well embedded culture of MDT working within this UK-based sample of healthcare practitioners, as well as recognition of the logistical problems of having multiple meetings.

The responses of our participants to questions about the amount of time spent at the MDT meeting are consistent with previous research carried out by the UK’s National Cancer Action Team (NCAT) [19]. 88% of respondents to the NCAT survey felt that a good MDT can save time later, which is comparable to our result of 68%. The NCAT survey reported that respondents felt strongly that effective MDT working resulted in improvements in clinical decision making, more coordinated patient care and improvements to the quality of care, findings that are consistent with the benefits in time saving given by our respondents.

Recent evidence has emerged that the arrangement of cases in an MDT meetings is associated with variation in the quality of decision-making [11]. Cases closer to the start of MDT meetings are associated with improved information presentation, improved teamworking and an increased chance of reaching a management decision – whereas cases later in the list fare worse in terms of the quality of their discussion. The results of the present study suggest that members favour prioritisation of cases on clinical grounds, which might give those at the start of meetings a favourable environment for discussion. Our findings are also consistent with the NCAT survey, which found that 78% of doctors and 78% nurses felt that cases should be grouped [5, 19].

The interpretation of the findings of this study is subject to certain limitations. The sample used in this study was small, consisting only of three professional groups. The study sample may not therefore be representative of MDT members in general. However, participants were recruited from national and regional forums, and as such represent those core MDT members who have contact with patients, from a range of locations throughout the UK. In addition, there is a possibility that as participation was opt-in in nature, those who responded are more favourably disposed to engaging in MDT working – this potential for self-selection bias is present in all surveys of this nature. Finally, the MDT members who have participated in this study are drawn from only urology MDTs, and therefore the results may not be generalizable to other tumour types. However, recent evidence suggests that consensus regarding what constitutes effective MDT working is high across common tumour types so some of our could also be applicable to other similar (i.e. high caseload) tumour types/teams [5]. Overall, replication of these results with a larger sample across tumour types other than urology will delineate the generalizability of the findings.

Although improving the quality of decision-making and standardising the decision-making process is a laudable aim, it is resource intensive and time consuming [20]. MDT meetings in other countries (often called tumour boards) are varied [3, 9, 15]. Indeed, Saini and colleagues provide an interesting insight into the variety of breast MDTs across 39 different countries, suggesting that there is much variation in the structure, case mix and decision-making processes across different countries around the world [16]. Freeman and colleagues found that the establishment of MDT services for patients with lung and oesophageal cnacer improved the efficacy and efficiency of treatment [9, 21]. In the UK, although cases of recurrence, and cases without curative intent (i.e. advanced disease) do not require discussion at a Specialist MDT meeting, these are cases that might benefit most from a MDT approach- improving information sharing, increasing expertise and streamlining referral between specialities. If such cases are to be brought to MDT meetings in the UK, then an already stretched service might buckle under the strain. Taking simple cases away from full MDT discussion and treating according to a pre-approved pathway may free up time and resources for complex cases – this is an important suggestion that stems from this study. Such an arrangement could be based on a combination of the clinical practice guidelines approved by the MDT (which may include the use of simple decision-making tools, such as *MDT-QuIC* [22]), with proposals for treatment/management circulated among MDT members before being proposed in the MDT meeting by the chair to ensure that such cases have met minimum requirements for information and any plans are carefully and fully recorded. A strategy such as this could provide quality assurance (comprehensive process of decision-making according to clinical practice guidelines, with the relevant information clearly documented, possibly using *MDT-QuIC* as a proforma) and quality control (decisions reviewed by the MDT, in addition to regular audits and the national peer review program) currently provided by full MDT discussion.

The cost of MDT working has not yet been adequately evaluated, and cost-effectiveness is even further from being defined. Estimates so far from the UK range from £14.10 ($23.12, €16.92) to £628.53 ($1,030.79, €754.24) per treatment plan [23]. Any assessment of the cost-effectiveness of MDT working will have to take account of upfront costs of MDT meetings, as well as any efficiencies (in terms of time or expense) that occur later in the treatment pathway, such as those identified by our respondents. Strategies to improve the efficiency of MDT meetings, either by streamlining simple cases, or by prioritising cases on clinical grounds to use time effectively, may also help to improve cost, as well as clinical effectiveness.

## Conclusions

There is an increasing body of evidence that MDT working in cancer care can improve the delivery of care for patients, as well as improving health outcomes. However, MDT working is time and resource intensive. This study is the first to explore which areas of urology MDT working healthcare professionals perceive to be valuable, and how MDT working might be improved in terms of effectiveness and efficiency. Such potential improvements will need to be tested empirically, but may translate into savings in both time and resources.

## Authors’ information

Authors Benjamin W Lamb and Rozh T Jalil are Joint first.

## Abbreviations

MDT: Multidisciplinary team.
